# Expression and Characterization of a Potent Long-Acting GLP-1 Receptor Agonist, GLP-1-IgG2σ-Fc

**DOI:** 10.1371/journal.pone.0156449

**Published:** 2016-05-27

**Authors:** Yi Yang, Fang Chen, Deyou Wan, Yunhui Liu, Li Yang, Hongru Feng, Xinling Cui, Xin Gao, Haifeng Song

**Affiliations:** 1 Anhui Medical University, Hefei, Anhui, China; 2 Research Center of Pharmacokinetics, Academy of Military Medical Sciences, Beijing, China; University of Lancaster, UNITED KINGDOM

## Abstract

Human GLP-1 (glucagon-like peptide-1) can produce a remarkable improvement in glycemic control in patients with type 2 diabetes. However, its clinical benefits are limited by its short half-life, which is less than 2 min because of its small size and rapid enzymatic inactivation by dipeptidyl peptidase IV. We engineered GLP-1-IgG2σ-Fc, a 68-kDa fusion protein linking a variant human GLP-1 (A8G/G26E/R36G) to a human IgG2σ constant heavy-chain. A stably transfected Chinese hamster ovary cell line was obtained using electroporation. Western blotting showed that the expressed protein was immunoreactive to both GLP-1 and IgG antibodies. GLP-1-IgG2σ-Fc stimulated insulin secretion from INS-1 cells in a dose- and glucose-dependent manner and increased insulin mRNA expression. The half-life of GLP-1-IgG2σ-Fc in cynomolgus monkeys was approximately 57.1 ± 4.5 h. In the KKAy mouse model of diabetes, one intraperitoneal injection of GLP-1-IgG2σ-Fc (1 mg/kg) reduced blood glucose levels for 5 days. A 4-week repeat-administration study identified sustained effects on blood glucose levels. Oral glucose tolerance tests conducted at the beginning and end of this 4-week period showed that GLP-1-IgG2σ-Fc produced a stable glucose lowering effect. In addition, KKAy mice treated with GLP-1-IgG2σ-Fc showed statistically significant weight loss from day 23. In conclusion, these properties of GLP-1-IgG2σ-Fc demonstrated that it represented a potential long-acting GLP-1 receptor agonist for the treatment of type 2 diabetes.

## Introduction

Type 2 diabetes mellitus (T2DM) is a progressive chronic disease characterized by hyperglycemia. The prevalence of this condition has increased in both developing and developed countries. The common classes of glucose-lowing agents include basal insulin, sulfonylureas, thiazolidinediones, dipeptidyl peptidase IV (DPP-IV) inhibitors, and glucagon-like peptide-1 (GLP-1) receptor agonists. Hypoglycemia is the major adverse effect of traditional anti-diabetic drugs. In contrast, preclinical studies indicated that the insulinotropic effect of GLP-1 was strictly glucose-dependent, which reduces the risk of hypoglycemia [1].

GLP-1 is an incretin hormone encoded by the proglucagon gene. This 30-amino acid protein exerts its biological effects by activating a G-protein-coupled receptor [2]. GLP-1 improves glycemic control in patients with T2DM by reducing their postprandial and fasting glucose levels. Its major pharmacological activities include the promotion of first- and second-phase insulin secretion, the suppression of glucagon activity under hyperglycemic conditions, and delaying the gastric emptying rate. Although GLP-1 provides effective weight control and blood glucose reduction, it is rapidly degraded *in vivo* and has a plasma elimination half-life (t_1/2_) of 2 min because of its rapid enzymatic degradation by DPP-IV and subsequent kidney clearance [3, 4]. The biologically active forms of GLP-1 are GLP-1(7–37) and GLP-1(7–36)NH_2_. DPP-IV preferably cleaves N-terminal Ala8-Gln9 dipeptide sequences, which converts GLP-1(7–37) and GLP-1(7–36)NH_2_ to the inactive peptides, GLP-1(9–37) and GLP-1(9–36)NH_2_. Extension of the t_1/2_ has become a key issue for research relating to GLP-1. DPP-IV inhibitors (sitagliptin, vildagliptin, saxagliptin, alogliptin, and linagliptin) and GLP-1 receptor agonists have therefore been developed to extend the t_1/2_. Clinical research confirmed that the use of DPP-IV inhibitors or GLP-1 receptor agonists significantly reduced fasting and postprandial blood glucose, HbA1c, and β-cell function. DPP-IV inhibitors and GLP-1 receptor agonists are mainly used to treat poorly controlled T2DM after sulfonylureas or thiazolidinediones have been employed. Their use is associated with a lower incidence of hypoglycemic events, and with good clinical safety and tolerability [5].

It is worth noting that more than 80% of people with T2DM are overweight or obese. There is robust research evidence indicating that obesity is a key determinant of insulin secretion and resistance to the effects of insulin. A modest reduction in body weight may therefore be beneficial for hyperglycemic control. According to the new guidelines of the American Diabetes Association and the European Association for the Study of Diabetes, GLP-1 receptor agonists are the only commonly used anti-diabetic agents shown to reduce body weight [6].

Five novel GLP-1 receptor agonists (exenatide, liraglutide, albiglutide, semaglutide, and dulaglutide) have been approved for the treatment of T2DM. Structural modification mainly includes cleavage site replacement [7] and the addition of a macromolecular protein or an aliphatic chain [8, 9]. These molecules can retain the biological activities of GLP-1, while showing a prolonged t_1/2_ [10]. The use of GLP-1 receptor agonists can cause gastrointestinal reactions and thyroid C cell proliferation [11, 12]. There is no research evidence for a correlation between these adverse reactions and the t_1/2_. However, antibodies and allergic reactions are frequently reported by research studies involving GLP-1 receptor agonists [13, 14]. To reduce immunogenicity and prolong t_1/2_, GLP-1-IgG2σ-Fc was constructed by fusing the human IgG2σ constant heavy-chain with a natural GLP-1 variant (A8G/G26E/R36G). The Fc region of IgG2σ has a t_1/2_ of 16 days [15]. This longer t_1/2_ is based on reduced renal clearance and FcRN-mediated receptor recycling [16]. IgG2σ is an Fc variant of IgG2 (V234A/G237A/P238S/H268A/V309L/A330S/P331S). In pre-clinical studies, IgG2σ showed minimal binding to Fcγ Rs and activation of immune responses, as compared to other previously well-characterized ‘muted’ Fc variants, including aglycosylated IgG1, IgG2m4, and IgG4 ProAlaAla (Dulaglutide is a fusion protein that includes a GLP-1 variant and IgG4 ProAlaAla Fc.) [15]. This GLP-1-IgG2σ-Fc fusion protein was therefore predicted to show a longer t_1/2_ and lower immune activity. We expressed the GLP-1-IgG2σ-Fc fusion protein and tested its bioactivity both *in vitro* and *in vivo* in order to investigate whether this represented a potential long-acting GLP-1 receptor agonist for the treatment of T2DM. GLP-1-IgG4-Fc was used as a positive control drug in this study instead of dulaglutide, which has yet to be marketed in China. GLP-1-IgG4-Fc, dulaglutide, and GLP-1-IgG2σ-Fc all include the same GLP-1 variant (A8G/G26E/R36G), fused to IgG4, IgG4 ProAlaAla (S228P/L234A/L235A), or IgG2σ (V234A/G237A/P238S/H268A/V309L/A330S/P331S), respectively. These features suggested that the t_1/2_, immunoactivity, and bioactivities of GLP-1-IgG4-Fc were likely to be more similar to those of dulaglutide than to those of liraglutide or exenatide.

## Materials and Methods

### Materials

Cell culture medium and serum were purchased from Invitrogen (Shanghai, China). GLP-1-IgG4-Fc fusion protein used as a positive control drug and was obtained from the Institute of Radiation Medicine, Academy of Military Medical Sciences (Beijing, China). The rat and mouse ultrasensitive insulin enzyme-linked immunosorbent assay (ELISA) kits were purchased from Alpco (Beijing, China). All other reagents, unless indicated, were purchased from Sigma-Aldrich (St Louis, MO, USA).

### Animals and Ethics Statement

This study was approved by the Institutional Animal Care and Use Committee at the animal center, Academy of Military Medical Sciences (IACUC: E20150602). Cynomolgus monkeys aged 3–4 years old (2.8–3.2 kg; animal numbers: 1009548, 1202501, and 1202473) were obtained from the Experimental Animal Center at the Beijing Sharing Institute of Biological Resources Co. Ltd. The monkeys were maintained in stainless steel cages (L × W × H: 800 × 700 × 750 mm) at a temperature of 20 ± 2°C, with 50–60% relative humidity, a 12-h light-dark cycle with artificial illumination from 0700 to 1900, and a room air exchange rate of 12 times/h. Animals had *ad libitum* access to water and fresh supplies of 300 g of standard monkey diet, while supplemental fruit and vegetables were offered twice daily. All monkeys were allowed to socialize by being housed in pairs during the day from approximately 09:00 to 15:00. Seasonal produce, seeds, and cereal were offered as supplements for environmental enrichment. During the experiment, the monkeys were housed individually with toys (such as mirrors, wooden trunks, and balls) and monitored daily by the animal care staff for any behavioral changes or illnesses. To minimize suffering, monkeys were lightly anesthetized with 5% inhaled isoflurane prior to their manipulation by veterinary staff. During the study period and prior to drawing blood, the monkeys were evaluated by a veterinary surgeon to determine whether the procedure should be carried out or discontinued. Since no significant trauma was involved in this study, only a brief muscle twitch (restricted to the stimulated limb) was expected. None of the cynomolgus monkeys was sacrificed and no complications resulting from manipulation were reported. Cynomolgus monkeys were placed back into their colony after the experiment and their use in other experiments was determined by the Institutional Animal Care and Use Committee at the animal center.

Male KKAy mice (10 weeks old) were purchased from Hua Fukang Biological Technology Co. Ltd. (Beijing, China) and maintained in a controlled environment (20 ± 2°C, 50–60% humidity) with a 12-h light-dark cycle (lights on at 07:00 and off at 19:00) and free access to water and food. During this study, blood samples were obtained by tail-vein prick. This method involves only slight trauma and was selected to minimize the potential suffering of these mice. The procedure would have been discontinued if the mice became stressed. All manipulations were performed under analgesia (2% inhaled isoflurane) to minimize suffering. Eighteen KKAy mice were used in this study and none of these became ill or died prior to its conclusion. At the end of the experiment, mice were euthanized by CO_2_ asphyxiation.

All animals were handled in accordance with the National Institutes of Health Guide for the Care and Use of Laboratory Animals. Every effort was made to minimize animal suffering and to reduce the number of animals used.

### Cell culture

INS-1 cells (passage 20–26) were obtained from the China Infrastructure of Cell Line Resources and maintained in RPMI 1640 medium containing 10% fetal bovine serum, 1 mM sodium pyruvate, 10 mM HEPES, and 25 nM β-mercaptoethanol at 37°C in an atmosphere of humidified air (95%) and CO_2_ (5%).

### Plasmid construction

A sequence encoding GLP-1-IgG2σ-Fc was designed using the nucleotide sequences of GLP-1 and IgG2σ and synthesized. This sequence was then digested by the restriction enzymes, *Hind*III and *Eco*RI (New England Biolabs, Ipswich, MA, USA), and inserted into the mammalian SGLs expression vector (obtained from the Research Center of Pharmacokinetics, Beijing, China). The recombinant SGLs-GLP-1-IgG2σ-Fc plasmid was verified by DNA sequencing (forward primer: 5ʹ-CAGGACCACGTCGTGCCAGT-3ʹ).

### Expression and purification of GLP-1-IgG2σ-Fc

This was conducted as described previously [17]. Chinese hamster ovary cells (passage 13–19) were obtained from the China Infrastructure of Cell Line Resources and expanded in CD-CHO complete medium (Invitrogen, Carlsbad, CA, USA) containing 8 mM glutamine. Stably expressing clones were obtained by electroporation with the SGLs-GLP-1-IgG2σ-Fc plasmid (Gene Pulser Xcell Electroporation System; BioRad, CA, USA). Highly expressing clones were selected based on SDS-PAGE and ELISA analyses using a horseradish peroxidase (HRP)-conjugated goat anti-human IgG monoclonal antibody at a 1:5000 dilution (catalog number 31413; Pierce, Rockford, IL, USA). Stable cell lines were cultured continuously for 12 days with rotation at 225 rpm at 37°C. The expression medium was then harvested and filtered. The GLP-1-IgG2σ-Fc fusion protein was purified from the medium by protein A affinity chromatography (Hi-Trap protein A column; GE Healthcare, Piscataway, NJ, USA). The retained protein was washed with 10 mM phosphate-buffered saline (PBS; 1 mL/min flow rate) and eluted with 100 mM sodium citrate-buffered saline (pH 3.0). All of these purification steps were carried out at 4°C. The sodium citrate-buffered saline was removed using an Amicon Ultra-4 ultrafiltration tube with a molecular weight cut-off of 10000 Da (Millipore, Bedford, MA, USA) at 4500 × *g* and 4°C for 45 min. The products were characterized by high-performance liquid chromatography, quantified using a bicinchoninic acid protein assay kit, and stored in 10 mM PBS (pH 7.4) at -80°C.

### Western blotting

Purified GLP-1-IgG2σ-Fc fusion protein was resolved by SDS-PAGE (10%) and transferred to nitrocellulose membranes. One membrane was probed with the HRP-conjugated goat anti-human IgG monoclonal antibody (1:5000; Pierce) for 60 min at 37°C. Another was probed with biotin-conjugated GLP-1 monoclonal antibody (1:5000, catalog number ABS033-10B-005; Thermo Fisher, Fremont, CA, USA) for 60 min at 37°C, followed by HRP-conjugated streptavidin (1:100000; catalog number AS-60668; Anaspec, San Jose, CA, USA) for 60 min at 37°C. After washing 6 times with PBS containing 0.05% Tween-20 (PBST), the protein bands were visualized using soluble 3,3ʹ,5,5ʹ-tetramethylbenzidine (TMB) (CWBIOTECH, Beijing, China).

### *In vitro* activity of GLP-1-IgG2σ-Fc

INS-1 cells were plated in 96-well assay plates at a concentration of 5 × 10^4^ cells/well. After a two-day maintenance period, 200 μL RPMI 1640 without glucose was added for 120 min to precondition the cells. This medium was then replaced with 200 μL RPMI 1640 supplemented with 2.2 mM or 16.8 mM glucose and GLP-1-IgG2σ-Fc (1 nM, 10 nM, or 100 nM) for 2 h. The amount of insulin released into the media was then evaluated using a rat ultrasensitive insulin ELISA kit.

Total RNA was also isolated from INS-1 cells incubated with GLP-1-IgG2σ-Fc using Trizol. For cDNA synthesis, 0.5 μg of total RNA was reverse-transcribed using a RevertAid First Strand cDNA Synthesis Kit (Thermo, USA). Real-time PCR (RT-PCR) was performed using a KAPA SYBR® FAST qPCR Kit and the fluorescent signal was detected by a Roche LightCycler® 96 system (Sweden) [18]. The following oligonucleotide primer pairs (forward and reverse) were used to amplify rat insulin: 5ʹ-CACCCAAGTCCCGTCGTGAAGT-3ʹ and 5ʹ-GATCCACAATGCCACGCTTCTG-3ʹ; and rat glyceraldehyde 3-phosphate dehydrogenase: 5ʹ-CCCACTCCTCCACCTTTGAC-3ʹ and 5ʹ-TCTTCCTCTTGTGCTCTTGC-3ʹ.

### Pharmacokinetics in cynomolgus monkeys

Pharmacokinetic studies of GLP-1-IgG2σ-Fc were carried out in adult male cynomolgus monkeys (n = 3). The monkeys were lightly anesthetized with 5% inhaled isoflurane, weighed, and placed on a heated primate chair to maintain normal body temperature. The heart rate, respiratory rate, blood pressure, and breathing pattern were continuously monitored. After fixation of the foreleg, blood (0.6 mL) was collected from the foreleg vein immediately pre-administration (time 0), and at 2, 4, 8, 12, 48, 72, 96, 192, 240, and 288 h after a single subcutaneous injection of GLP-1-IgG2σ-Fc (0.1 mg/kg) using a retained needle and a 1-mL syringe. After the collection of blood, the animal was returned to the recovery cage and monitored until it was able to sit up. Plasma samples were obtained by centrifugation and stored at -70°C in polyethylene tubes containing 10 μL of DPP-IV inhibitor (Millipore, Milford, MA, USA). The GLP-1-IgG2σ-Fc levels were determined by sandwich ELISA, using a mouse anti-GLP-1 monoclonal antibody (1:500; catalog number ab121086; Abcam, Cambridge, UK), a secondary HRP-conjugated goat anti-human IgG monoclonal antibody (1:20000; Pierce), and TMB as the chromogenic reagent. Standard curves were prepared for GLP-1-IgG2σ-Fc in cynomolgus monkey plasma. The ELISA assay range was approximately 0.66–1000 ng/mL. Concentrations of GLP-1-IgG2σ-Fc were calculated using Watson LIMS v.7.3.0.01 (Thermo Scientific Inc.). Pharmacokinetic data were analyzed by noncompartmental methods using WinNonLin version 5.2.1 (Pharsight Inc., Mountain View, CA, USA).

### Oral glucose tolerance test (OGTT)

Eighteen KKAy mice (10 weeks old; 34–37 g) were acclimated for 3 days and fasted overnight (12 h) before they were randomly divided into three groups (n = 6/group).These groups received GLP-1-IgG2σ-Fc (1 mg/kg), GLP-1-IgG4-Fc (1 mg/kg), or saline by intraperitoneal injection 30 min prior to the OGTT. Glucose was dissolved in saline and administered by oral gavage at a dose of 2 g/kg. Blood samples were obtained by tail-vein prick at pre-determined time points (-30, 0, 10, 20, 30, 60, and 90 min) and glucose levels were measured using a glucose meter (One Touch®Ultra; LifeScan, NJ, USA) [19].

### Acute and long-term pharmacodynamics

After a 3-day acclimatization period, 10-week-old male KKAy mice were injected intraperitoneally with GLP-1-IgG2σ-Fc (1 mg/kg), GLP-1-IgG4-Fc (1 mg/kg), or saline (200 μL). Blood glucose levels were measured using a glucose meter at 0, 1, 2, 3, 4, 5, 6, and 7 days (08:00).

For the long-term study, KKAy were acclimatized for 3 days prior to intraperitoneal injection of GLP-1-IgG2σ-Fc (1 mg/kg), GLP-1-IgG4-Fc (1 mg/kg), or saline (200 μL) once every three days for 4 weeks. Blood glucose was measured using a glucose meter before these injections. Body weight and food intake were recorded every day. At the end of this treatment period, the mice had 10 drug administration-free days before they were subjected to the OGTT test described above. Blood samples were obtained by tail-vein prick at pre-determined time points (-30, 0, 10, 20, 30, 60, and 90 min) and glucose levels were measured using a glucose meter. Blood samples (obtained at -30 and 30 min) were centrifuged at 3000 rpm for 5 min at 4°C to isolate plasma. Insulin levels were evaluated using a mouse ultrasensitive insulin ELISA kit (Alpco, Beijing, China).

### Statistical analysis

Concentrations of GLP-1-IgG2σ-Fc and insulin were calculated using a four-parameter algorithm. The area under the blood glucose concentration-time curve (AUC) in the OGTT was calculated using the following algorithm: AUC (mM・120 min) = (BG_-30_ + BG_0_) × 15 + (BG_0_ + BG_10_) × 5 + (BG_10_ + BG_20_) × 5 + (BG_20_ + BG_30_) × 5 + (BG_30_ + BG_60_) × 15 + (BG_60_ + BG_90_) × 15. BG_-30_, BG_0_, BG_10_, BG_20_, BG_30_, BG_60_, and BG_90_ represent the blood glucose levels at -30, 0, 10, 20, 30, 60, and 90 min, respectively. All data were expressed as means ± the standard error of the mean (SEM), and differences between groups in assays were determined by one-way ANOVA followed by Tukey’s post hoc multiple comparison test using GraphPad Prism 5 software. A p-value of less than 0.05 was regarded as statistically significant.

## Results

### Construction, expression, and purification of GLP-1-IgG2σ-Fc

**Fig 1:**
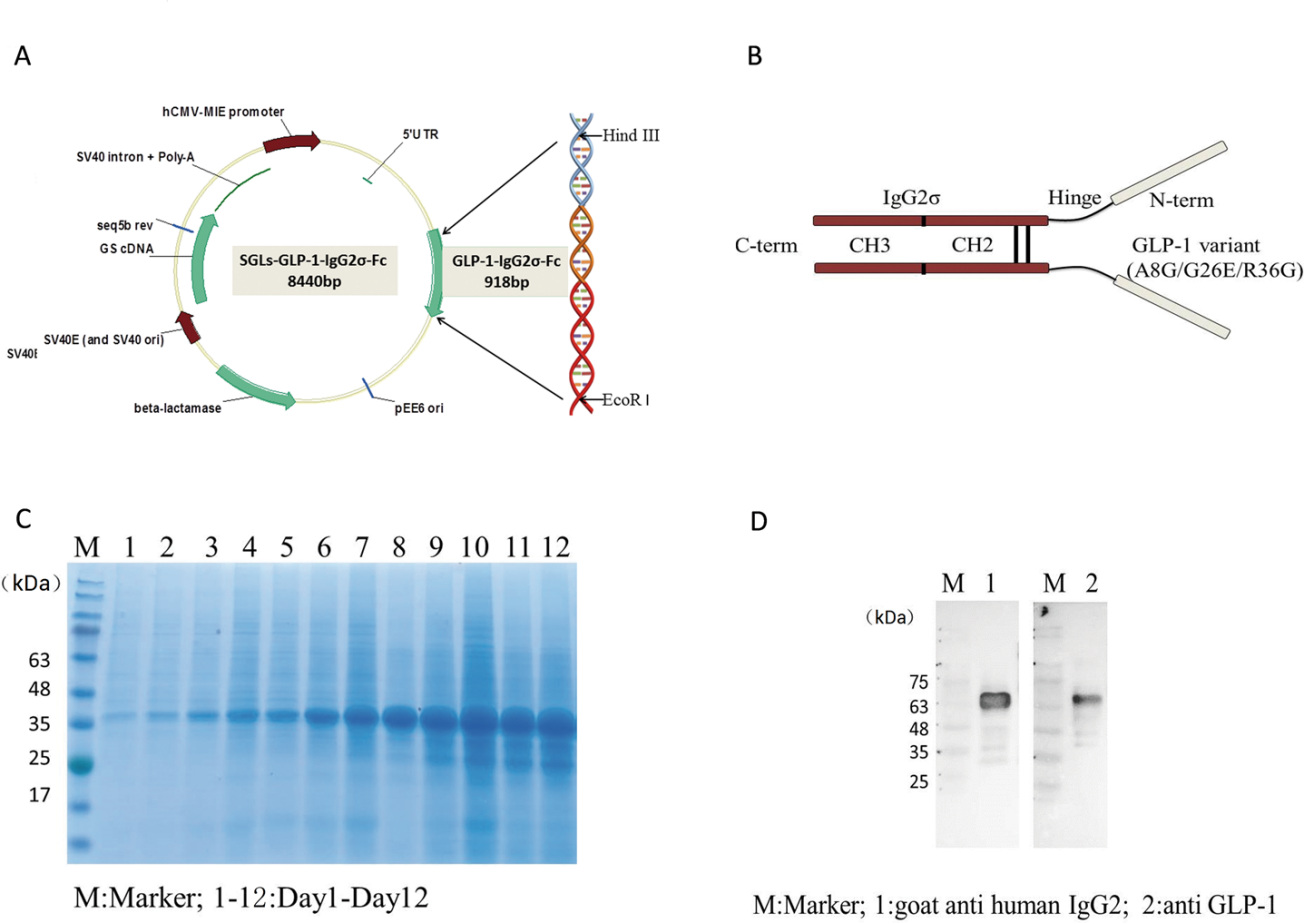
Generation of the GLP-1-IgG2σ-Fc expression vector. (A) DNA encoding GLP-1-IgG2σ-Fc was synthesized and inserted into a mammalian expression vector, SGLs. (B) GLP-1-IgG2σ-Fc comprises a pair of human GLP-1 variants (A8G/G26E/R36G) and a human IgG2σ constant heavy-chain, with a molecular mass of 68 kDa. (C) SDS-PAGE analysis of cell culture medium indicated that stably transfected cells secreted the highest amount of GLP-1-IgG2σ-Fc on day 12. (D) Western blotting analysis confirmed that the expressed 68-kDa protein showed good antigenicity to both GLP-1 and human IgG.

DNA encoding GLP-1-IgG2σ-Fc (918 bp) was synthesized and inserted into the mammalian SGLs expression vector (7956 bp) (Fig 1A). DNA sequencing demonstrated that the insertion sequence was correct. The expressed GLP-1-IgG2σ-Fc protein included a DPP-IV-protected GLP-1 variant (A8G/G26E/R36G) linked to the human IgG2σ constant heavy-chain by a peptide linker (GGGSGGGSGGGS) (Fig 1B) [20]. Analysis of CHO-S culture medium by SDS-PAGE indicated that these cells secreted the highest level of GLP-1-IgG2σ-Fc on day 12 (Fig 1C). Western blotting analysis confirmed that the expressed protein showed good antigenicity to both GLP-1 and human IgG antibodies and had an apparent molecular weight of 68 kDa (Fig 1D). After one-step purification using protein A affinity chromatography, this expressed GLP-1-IgG2σ-Fc was used in subsequent experiments.

### *In vitro* activity of GLP-1-IgG2σ-Fc

**Fig 2:**
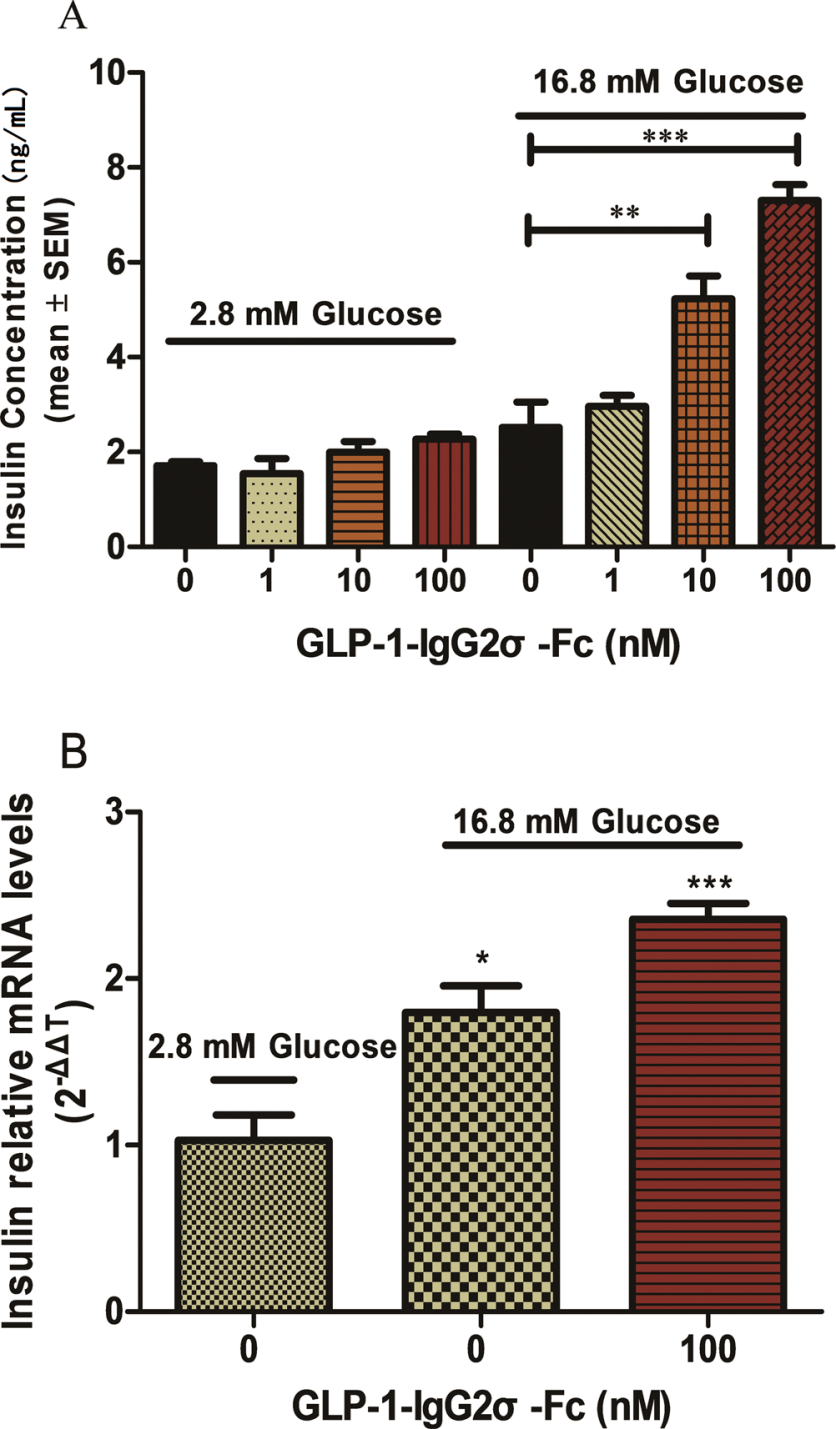
Insulin synthesis and secretion in INS-1 cells. (A) INS-1 cells were plated in 96-well plates with glucose-free RPMI 1640 for 120 min before media supplemented with the indicated concentrations of glucose and GLP-1-IgG2σ-Fc were added for 2 h. Insulin levels were measured by ELISA. (B) Insulin mRNA levels in the INS-1 cells treated as indicated were determined by RT-PCR. Values are means ± SD (n = 6). All INS-1 cell experiments were repeated for four times. A representative result of multiple independent experiments is presented. *p < 0.05 versus vehicle-treated cells in 16.8-mM glucose media.

Tests of glucose-induced insulin secretion were performed using INS-1 cells exposed to various concentrations of GLP-1-IgG2σ-Fc. As shown in Fig 2A, GLP-1-IgG2σ-Fc stimulated insulin secretion in a glucose-dependent manner. In cells incubated with medium containing 2.8 mM glucose, there were no statistically significant differences between each group. However, cells incubated with medium containing 16.8 mM glucose secreted significantly more insulin in the presence of 10 nM (5.2 ± 0.4 ng/mL) and 100 nM (7.3 ± 0.3 ng/mL) GLP-1-IgG2σ-Fc, as compared with control cells (2.5 ± 0.5 ng/mL) (p < 0.05). To investigate this effect on insulin secretion further, mRNA was isolated from the INS-1 cells exposed to 16.8 or 2.2 mM glucose and 100 nM GLP-1-IgG2σ-Fc and insulin mRNA expression was evaluated by RT-PCR (Fig 2B). Cells exposed to 16.8 mM glucose in the presence of 100 nM GLP-1-IgG2σ-Fc showed a significant increase in insulin mRNA level, as compared to those exposed to 16.8 and 2.2 mM glucose only (without GLP-1-IgG2σ-Fc).

### GLP-1-IgG2σ-Fc has an extended t_1/2_ and produced sustained glucose reduction

**Fig 3:**
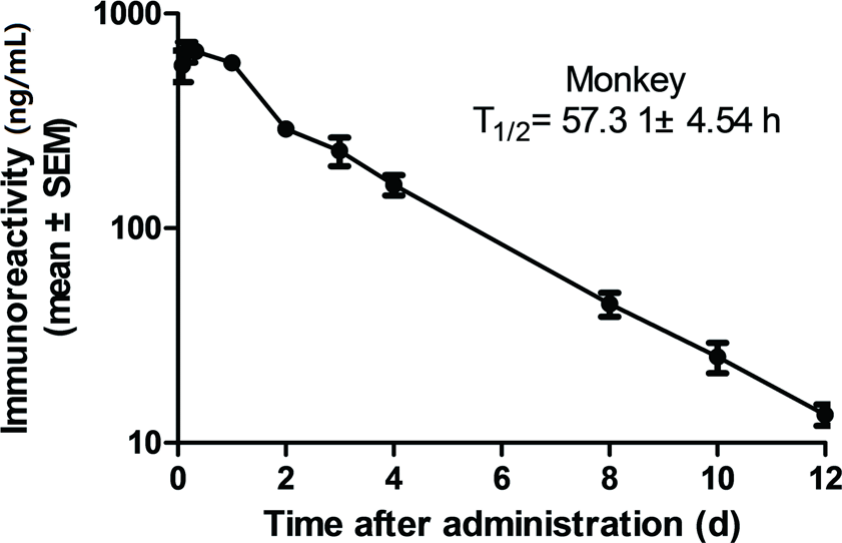
Pharmacokinetics in cynomolgus monkeys. Plasma fusion protein levels were determined by ELISA following administration of 0.1 mg/kg GLP-1-IgG4-Fc.

The pharmacokinetics of GLP-1-IgG2σ-Fc were studied in cynomolgus monkeys after a single dose of 0.1 mg/kg. The t_1/2_ of GLP-1-IgG2σ-Fc was approximately 57.1 ± 4.5 h and it could be detected for more than 14 days after a single injection (Fig 3 and Table 1).

**Table 1:** Pharmacokinetic parameters in cynomolgus monkeys.

	C_max_ (ng/mL)	T_max_ (h)	AUC_0-∞_(h·ng/mL)	t_1/2_ (h)	CL/F (mL/h/kg)	Vd (mL/kg)	MRT (h)
Monkey	701.0 ± 52.2	6.7 ± 1.3	48353.2 ± 3228.6	57.1 ± 4.5	2.1 ± 0.1	172.9 ± 22.3	68.6 ± 2.0

Pharmacokinetic parameters (mean ± SEM) were calculated using the plasma levels of GLP-1-IgG2σ-Fc in three cynomolgus monkeys at each time-point following administration of 0.1 mg/kg. C_max_, maximum plasma concentration; T_max_, time of maximum plasma concentration; AUC, area under the curve; t_1/2_, elimination half-life; CL/F, clearance; Vd, volume of distribution; MRT, mean residence time.

**Fig 4:**
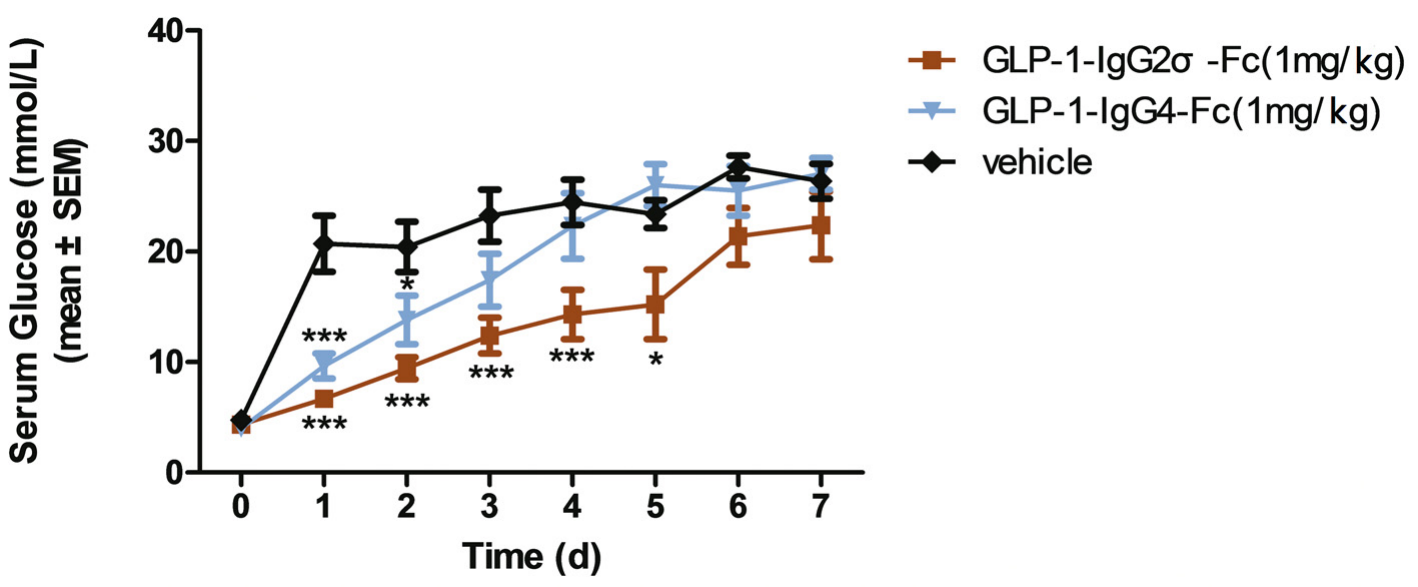
Acute pharmacodynamics. KKAy mice received a single intraperitoneal injection of GLP-1-IgG2σ-Fc (1 mg/kg), GLP-1-IgG4-Fc (1 mg/kg), or saline. Blood glucose levels were measured at the indicated time-points. *p < 0.05; **p < 0.01; ***p < 0.001, versus the vehicle-treated group.

The blood glucose level in KKAy mice showed a sustained reduction after a single dose of 1 mg/kg GLP-1-IgG2σ-Fc (Fig 4). Mice treated with GLP-1-IgG2σ-Fc showed significantly lower levels of postprandial glucose from days 1 to 5, as compared to the vehicle group. The GLP-1-IgG4-Fc-treated group only showed a statistically significant hypoglycemic effect on the first two days post-administration. Based on this result and on pre-clinical research involving dulaglutide, the administration frequency was set to three days [21].

### GLP-1-IgG2σ-Fc reduced postprandial glucose and body weight in KKAy mice

**Fig 5:**
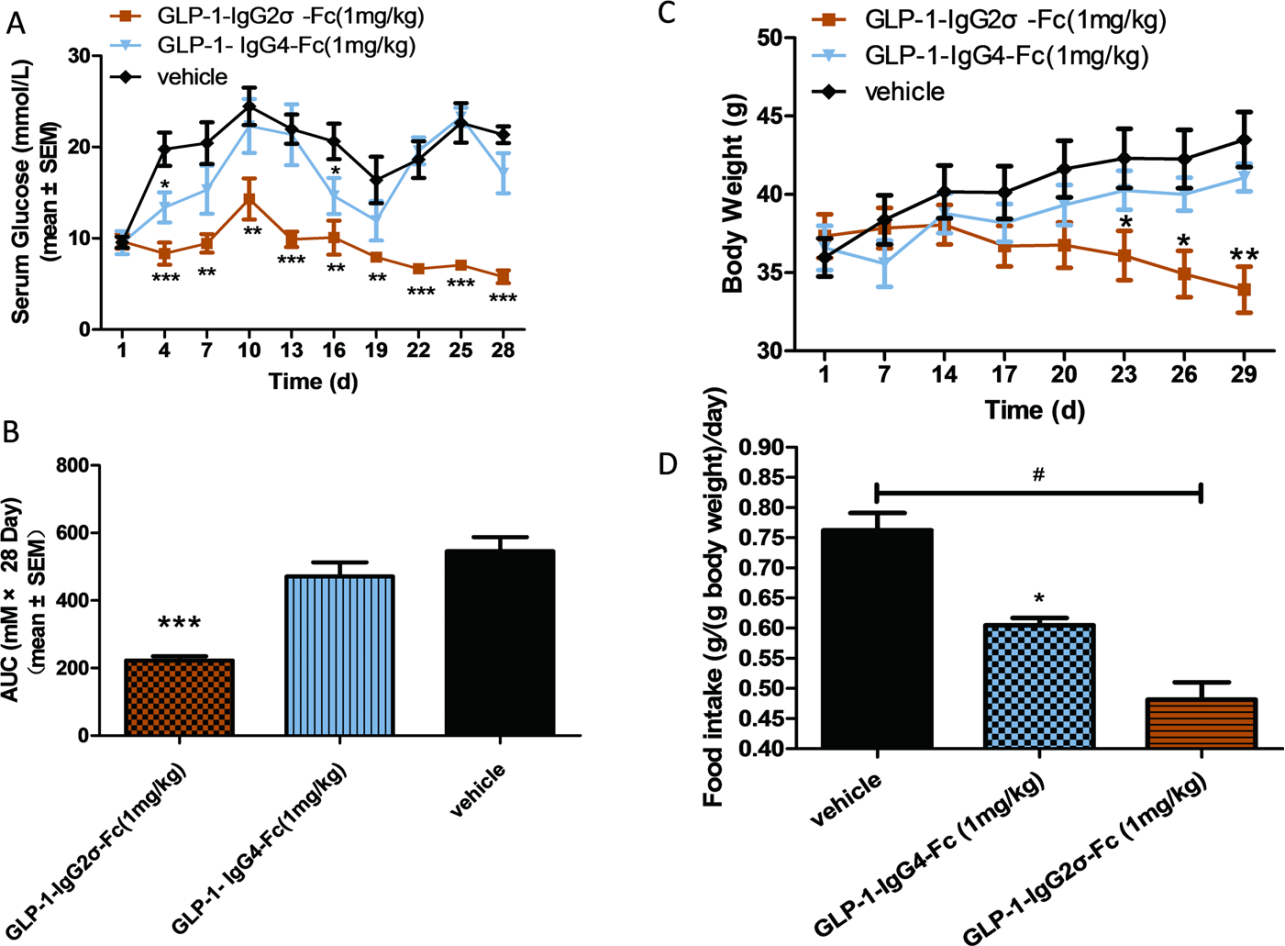
Long-term pharmacodynamics. (A) Blood glucose was measured prior to the administration of the indicated treatments to KKAy mice every three days for 4 weeks. (B) The area under the curve (AUC) of the data presented in (A). (C) Mouse body weights were recorded every day. (D) Food intake of each group. *p < 0.05; **p < 0.01; ***p < 0.001, versus the vehicle-treated group; ^#^p < 0.05, versus the GLP-1-IgG4-Fc-treated group.

KKAy mice were treated with intraperitoneal GLP-1-IgG2σ-Fc once every three days for 4 weeks. During this period, GLP-1-IgG2σ-Fc significantly reduced the plasma glucose levels (Fig 5A). The AUC from days 1 to 28 was significantly larger in the vehicle-treated group than in the 1 mg/kg GLP-1-IgG2σ-Fc-treated group (p < 0.001; Fig 5B). KKAy mice treated with GLP-1-IgG2σ-Fc had a significantly lower body weight from day 23, while the group treated with GLP-1-IgG4-Fc showed no significant difference, as compared to the vehicle group (Fig 5C). The vehicle group consumed ~1.5-fold more food than the GLP-1-IgG2σ-Fc group and ~1.3-fold more food than the GLP-1-IgG4-Fc group. In addition, the GLP-1-IgG2σ-Fc group consumed significantly less food than the GLP-1-IgG4-Fc group.

**Fig 6:**
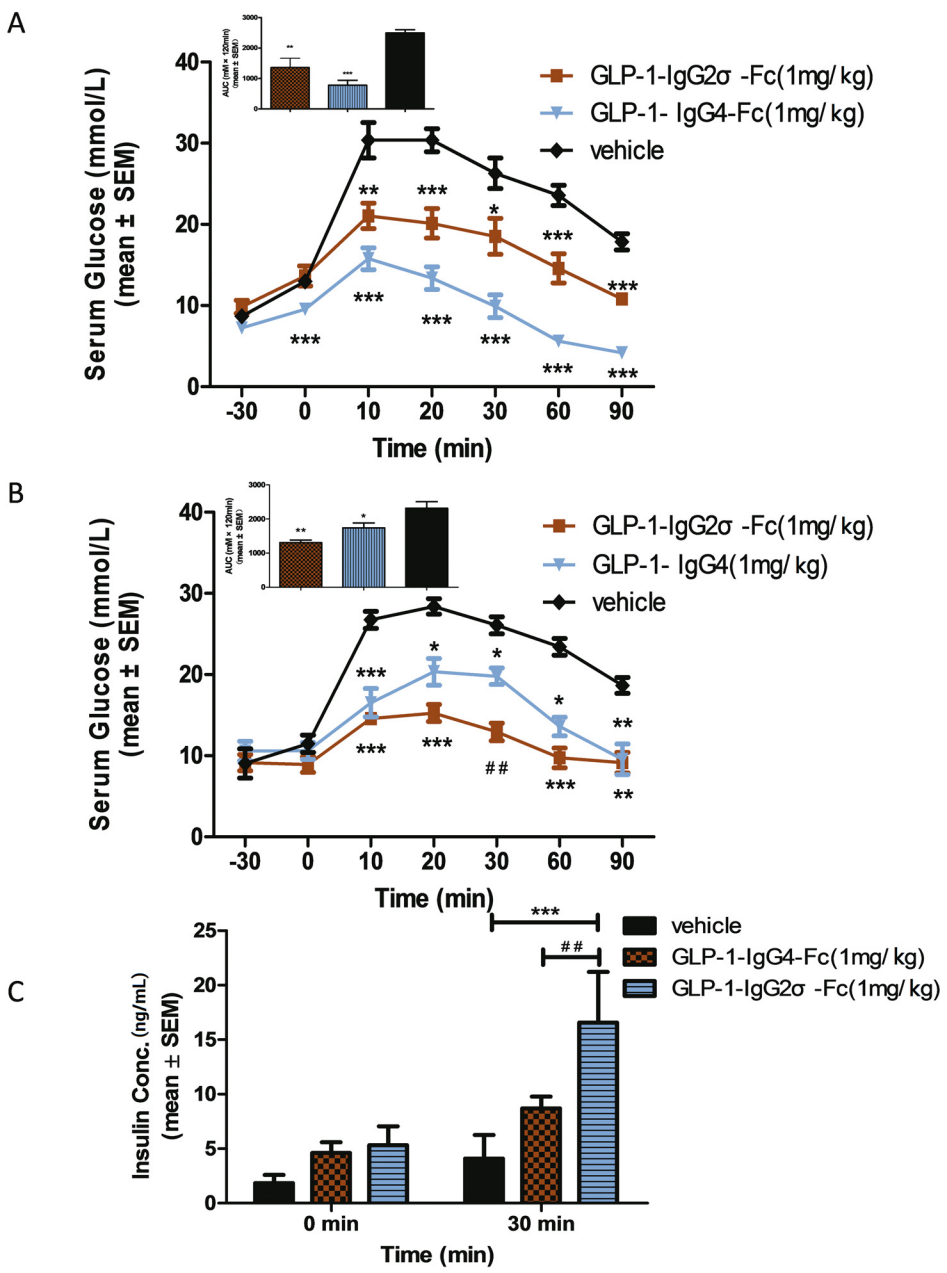
OGTT in KKAy mice. (A) OGTT in KKAy mice at the beginning of the 4-week treatment period. Glucose (2 g/kg) was administered 30 min before injection of GLP-1-IgG2σ-Fc (1 mg/kg), GLP-1-IgG4-Fc (1 mg/kg), or saline (200 μL). The area under the curve (AUC) for the glucose levels is shown in the upper left panel. (B) OGTT in KKAy mice at the end of the 4-week treatment period. The AUC for the glucose levels is shown in the upper left panel. (C) Insulin levels were tested at 0 and 30 min during the OGTT test; *p < 0.05; **p < 0.01; ***p < 0.001 versus the vehicle-treated group.

OGTT tests were performed in KKAy mice at the beginning and end (10 days after the last dose) of a 4-week treatment with GLP-1-IgG2σ-Fc, GLP-1-IgG4-Fc, or saline to investigate the effects on postprandial glucose levels. Compared with the vehicle group, mice treated with either GLP-1-IgG4-Fc or GLP-1-IgG2σ-Fc showed reduced glucose levels. Calculation of the AUC showed that mice treated with GLP-1-IgG4-Fc showed a reduction of ~61.5% before treatment and of ~24.9% after treatment. GLP-1-IgG2σ-Fc produced a greater hypoglycemic effect after treatment, with a reduction of ~31.2% before treatment and ~43.5% after treatment (Fig 6A–6D). Insulin levels were tested after 4 weeks of treatment to further investigate the effect of GLP-1 fusion proteins in KKAy mice. The groups treated with GLP-1-IgG4-Fc and GLP-1-IgG2σ-Fc showed no significant improvements in basal insulin levels prior to gastric perfusion of glucose. As compared with vehicle-treated mice, those treated with GLP-1-IgG4-Fc or GLP-1-IgG2σ-Fc showed significant improvements in insulin secretion at 30 min (Fig 6C).

## Discussion

GLP-1-Fc fusion proteins can be used to produce molecules that retain the glycemic control properties of GLP-1, with longer t_1/2_ values. We successfully engineered GLP-1-IgG2σ-Fc, a 68-kDa fusion protein linking variant human GLP-1 (A8G/G26E/R36G) with a human IgG2σ constant heavy-chain. We expressed this in a eukaryotic expression system because this is the only established system that is suitable for the expression of complex recombinant proteins with human-like glycoforms that are bioactive in humans [22]. The protein yield of 1.2 g from 1 L culture medium, continuously cultured for 12 days (data not shown), confirmed that this mammalian expression system was a highly efficient producer of GLP-1-IgG2σ-Fc.

Consistent with our previous observations using native GLP-1 and GLP-1 receptor agonists, incubation of INS-1 cells with GLP-1-IgG2σ-Fc resulted in concentration-dependent effects on insulin release [23]. Further mRNA analyses of these cells indicated that GLP-1-IgG2σ-Fc also increased insulin synthesis. This confirmed that the method used to purify the expressed GLP-1-IgG2σ-Fc did not inhibit its bioactivity. The results also confirmed that GLP-1-IgG2σ-Fc stimulated insulin secretion in a glucose-dependent manner.

GLP-1-IgG4-Fc (comprising native GLP-1 fused with IgG4) was used as a positive control in the present *in vivo* study. The t_1/2_ of GLP-1-IgG4-Fc in cynomolgus monkeys was approximately 37.5 ± 4.9 h (data not shown), while the t_1/2_ of GLP-1-IgG2σ-Fc was 57.1 ± 4.5 h. This represented a significant improvement over native GLP-1 (t_1/2_ < 5 min). Other GLP-1 agonists have shown t_1/2_ values of 51.6 ± 3.2 h in monkeys (dulaglutide), 11–13 h in humans (liraglutide), and 2.4 h in humans (exenatide BID). Due to species differences, dulaglutide (a human GLP-1 variant fused with a human IgG4 variant) showed a longer t_1/2_ in humans than in monkeys (120 h versus 51.6 ± 3.2 h, respectively) and further research will be required to determine whether GLP-1-IgG2σ-Fc also has a longer t_1/2_ in humans.

GLP-1 and GLP-1 receptor agonists improve glycemic control in T2DM by reducing postprandial glucose levels [24]. The present study used ten-week-old male KKAy mice as an *in vivo* model of T2DM because these mice develop non-insulin-dependent diabetes mellitus spontaneously at the age of 12 weeks [25–27]. Although KKAy, db/db, and ob/ob mice are all used as genetic mouse models of T2DM, KKAy mice were the least expensive of these and were readily available. GLP-1-IgG2σ-Fc produced a more sustained hypoglycemic effect in KKAy mice than did GLP-1-IgG4-Fc. An intraperitoneal injection of GLP-1-IgG2σ-Fc (1 mg/kg) reduced blood glucose levels for 5 days, while GLP-1-IgG4-Fc affected this for 2 days. Based on this observation and on previous studies of dulaglutide, we elected to administer these treatments once every three days during the present long-term pharmacodynamics study. Our findings suggested that GLP-1-IgG2σ-Fc produced a stable hypoglycemic and insulin secretion effect during the treatment period. Notably, the drug resistance observed in animals treated with GLP-1-IgG4-Fc was not seen in those treated with GLP-1-IgG2σ-Fc. Bioactivity is often reduced by neutralizing antibodies, which can be generated at different levels, depending on the immunogenicity of the protein drug [28]. Several studies of GLP-1 receptor agonists have reported the formation of antibodies and exenatide treatment was associated with antibody generation in 74% of patients [29]. Liraglutide, which shares 97% of its amino acid sequence with the endogenous human protein, also generated antibodies in 4–13% of patients [30, 31]. The variation present in IgG2 completely eliminates its affinity for FcγRs and the C1q complement protein, which induce antibody-dependent cellular cytotoxicity and phagocytosis, as well as complement-dependent cytotoxicity. These ‘silent’ properties of IgG2σ render it superior to IgG4 for use in therapeutic non-immunostimulatory fusion proteins. In addition, the GLP-1 variant used in the present study is a close structural homolog of native human GLP-1, with three amino acid substitutions (A8G/G26E/R36G) that confer protection from DPP-IV cleavage. The longer t_1/2_ and stable glucose-lowering effects observed in our *in vivo* study indicated that GLP-1-IgG2σ-Fc did not trigger a significant level of antibody production and was not metabolized rapidly. This suggested that GLP-1-IgG2σ-Fc may be appropriate for therapeutic application as a GLP-1 receptor agonist.

Their effect on weight loss is another important property of GLP-1 receptor agonists [32]. In the present pharmacodynamic study, 4-week treatment with GLP-1-IgG2σ-Fc resulted in significant weight loss from day 23, as compared to the vehicle group. The food intake result also supported this conclusion and indicated that GLP-1-IgG2σ-Fc may reduce weight by inhibiting feeding. However, it is not possible to extrapolate these pre-clinical findings to infer effects in humans. For example, dulaglutide includes the same GLP-1 variant as GLP-1-IgG2σ-Fc and produces significant weight reduction in db/db mice, but not in humans.

## Conclusions

The hypoglycemic effects of GLP-1-IgG2σ-Fc were sustained for longer than those of GLP-1-IgG4-Fc in KKAy mice. Pharmacokinetic and pharmacodynamic analyses of GLP-1-IgG2σ-Fc confirmed that it had a prolonged t_1/2_ and could reduce body weight. These superior features indicated that GLP-1-IgG2σ-Fc could provide a potential long-acting GLP-1 receptor agonist for the treatment of T2DM.
